# Evaluation of an infection control link nurse program: an analysis using the RE-AIM framework

**DOI:** 10.1186/s12913-023-09111-5

**Published:** 2023-02-09

**Authors:** Mireille Dekker, Irene P. Jongerden, Martine G. Caris, Martine C. de Bruijne, Christina M. J. E. Vandenbroucke-Grauls, Rosa van Mansfeld

**Affiliations:** 1 Department of Medical Microbiology and Infection Prevention, Amsterdam UMC, Vrije Universiteit Amsterdam, De Boelelaan 1118, 1081 HV Amsterdam, The Netherlands; 2 Department of Public and Occupational Health, Amsterdam UMC, Vrije Universiteit Amsterdam, Amsterdam Public Health Research Institute, Amsterdam, The Netherlands; 3 Department of Clinical Medicine – Department of Clinical Epidemiology, Aarhus University, Aarhus, Denmark

**Keywords:** Quality improvement, Patient safety, Nursing practice, Nosocomial infections, Infection prevention

## Abstract

**Background:**

Important elements of programs that train and support infection control link nurses (ICLN) are the engagement of stakeholders, support from hospital and ward management and a structure for iterative improvement. The effects of programs, that combine all these elements, are unknown. We evaluated such a comprehensive program to explore its impact on link nurses and infection prevention practices and routines.

**Methods:**

We used the RE-AIM framework, a robust, evidence-based framework within the field of Implementation Science, to evaluate the impact of our ICLN training and support program. We used a mixed methods approach and organized the outcomes along its five dimensions: Reach, Effectiveness, Adoption, Implementation and Maintenance.

**Results:**

Between 2014 and 2018, on average 91% of the inpatient wards and 58% of the outpatient clinics participated in the program (Reach) and impacted guideline adherence in inpatient wards. Link nurses felt engaged and empowered, and perceived their contribution to these results as pivotal. Ward managers confirmed the value of ICLN to help with implementing IPC practices (Effectiveness). The program was adopted both at the hospital and at the ward level (Adoption). Based on ongoing evaluations, the program was adapted by refining education, training and support strategies with emphasis on ward specific aspects (Implementation). The ICLN program was described as a key component of the infection prevention policy to sustain its effects (Maintenance).

**Conclusions:**

Our infection control link nurse program helped ICLN to improve infection prevention practices, especially in inpatient wards. The key to these improvements lay within the adaptability of our link nurse program. The adjustments to the program led to a shift of focus from hospital goals to goals tailored to the ward level. It allowed us to tailor activities to align them with the needs specific to each ward.

**Supplementary Information:**

The online version contains supplementary material available at 10.1186/s12913-023-09111-5.

## Background

Infection control link nurses (ICLN) serve as role models in providing safe care; they are trained to monitor infection prevention-related issues on their ward and to inform and facilitate their peers so they can improve their clinical practice [1, 2]. Since their first introduction in the 1980’s, link nurses liaised between the clinical wards and the epidemiology department for the surveillance of health care associated infections [3]. Nowadays, activities of link nurses are supported by dedicated programs that seek to improve the dissemination and implementation of infection prevention and control guidelines [3, 4]. This approach to implementation is used in hospitals all over the world [3]. Key element of these programs is the education of ICLN by infection control practitioners. Infection control practitioners are members of the Infection Prevention and Control team; they are experts in the prevention and control of healthcare associated infection. Link nurse programs are often developed locally, which means there is a wide variety in content and in ways these programs are developed, implemented, and evaluated. Although their success depends on contextual factors and their specific use, proven effective elements to start and maintain link nurse programs are the engagement of stakeholders, support from hospital and ward management, and a structure for iterative improvement (e.g. plan-do-check-act (PDCA) cycle) [3–8]. In addition, programs that provide training in implementation skills on top of education on infection prevention topics are rated higher by infection control practitioners [7, 9]. At the individual level, as authority is perceived to be essential to fulfill the ICLN role, participation of more experienced nurses is preferred [1, 10, 11]. However, there is limited knowledge on whether ICLN programs that combine these elements are indeed more successful.

To improve the understanding of factors for success and the reporting of outcomes of ICLN programs, evaluation theories, models or frameworks can be used [12]. RE-AIM is a robust, evidence-based framework that facilitates the description of all relevant aspects of programs in real-world settings, providing valuable information on their impact, including barriers and facilitators [13, 14]. It comprises five dimensions: Reach, Effectiveness, Adoption, Implementation and Maintenance (https://re-aim.org/). RE-AIM has proven to be applicable in the evaluation of prevention programs; it can help to understand the efficacy and effectiveness of these programs implemented in real-world settings [15–17]. Most studies that used the RE-AIM framework have used descriptive and quantitative data for their evaluations. A more recent paper researchers emphasis the need to assess its dimensions by using qualitative data in order to enhance the understanding of the’how’ and ‘why’ [14].

The objective of the present study was to evaluate an ICLN program that was initiated in 2014 by the infection prevention and control department of our university hospital, on all dimensions of the RE-AIM framework. We aimed to 1) explore how it impacted link nurses, infection prevention and control (IPC) practices and routines specific to our university hospital, and 2) contribute to the body of knowledge on how to initiate and sustain an ICLN program using essential elements from literature.

## Methods

### Study design

We used a mixed methods approach to evaluate the first four years of our infection control link nurse program and explored its impact on link nurses, infection prevention practices and the routines in our academic hospital. We integrated the descriptive, quantitative and qualitative data to yield more complete evidence in order to enhance deeper understanding of this complex implementation process We applied the RE-AIM framework to structure our findings in order to evaluate this program on its different dimensions. We followed the Standards for Reporting Implementation Studies (StaRI) and the Revised Standards for Quality Improvement Reporting Excellence (SQUIRE 2.0) to report the findings of this study [18, 19]. The completed StaRI checklist can be found in Additional file 1.

### Ethical considerations

The need for approval for this study was waived by the Medical Ethical Committee at Amsterdam UMC, Vrije Universiteit Amsterdam (2018.485).

### Setting

The Amsterdam University Medical Centers, location VUmc is a 700-bed university hospital with inpatient wards and outpatient clinics. The infection prevention and control team is part of the department of Medical Microbiology and Infection Prevention. In 2014, this team initiated an ICLN program as part of a larger project targeting several antimicrobial stewardship and infection control issues. The ICLN program was designed to form closer alliances with nurses from various wards to create awareness of infection prevention and to promote and implement safe practices.

### Intervention

The managers of all inpatient wards were requested to recruit at least one link nurse. Link nurses volunteered and were then selected by their ward manager. ICLN from outpatient clinics were welcome to join the program but were only officially recruited and appointed from 2016. Link nurses were educated by infection control practitioners on infection prevention topics, who also registered meeting attendance. After each meeting all ICLN received an email with tools (e.g., PowerPoint, Quick Scan format, newsletter, implementation tip sheet) to implement the infection prevention topics that were discussed during that meeting. Link nurses received a certificate of attendance per meeting to enter into their quality register portfolio. When ICLN did not attend, the infection control practitioner sent an email to the link nurse and ward management, offering support for the implementation. In 2018, with the help of ward-based annual plans and evaluations, iterative PDCA cycles were introduced at the ward level, to address ward specific issues. The ward manager was responsible for the backup and support of the ICLN. The infection control practitioner provided practical input and coached ICLN in effectuating these plans. At the hospital level the PDCA-cycles were operationalized as follows: Outcomes of the program (e.g., meeting attendance per ward, ward-based plans and audit results) were reported to the board of nursing directors on a yearly basis, the first two years via oral presentations, after 2016 through written reports.

The ICLN program was planned per year, based on outcomes of the program and on audit results.The ICLN program originally consisted of topics that were clustered in one theme per year.. Themes were, in order: general precautions, isolation precautions, cleaning and disinfection, and separation of clean and contaminated work areas. To support dissemination, ICLN were trained to perform quick scans on the selected topics and to report their findings to their peers. In 2017 we added training in implementation skills and simulation training to the program. From this time onward, link nurses were also encouraged to share their best practices during festive end-of -year lunch meetings by means of a poster or oral presentation.

### Data collection

We used routinely collected data, such as surveillance data, attendance data and meeting minutes, questionnaires, and hospital policy and project documents, originating from initial planning and start, followed by monitoring of the program from January 2014 to December 2018. We also used data from semi-structured face-to-face interviews which were part of a qualitative study with the aim to explore the experiences of infection control link nurses regarding their role, which was published elsewhere [2]. Table 1 outlines how we defined and measured the five RE-AIM dimensions and which data sources we used.

**Table 1:** RE-AIM elements, measurement methods and outcome measures

RE-AIM dimension	Definition operationalized for this study	Outcome measures	Data Sources
**Reach**	The absolute number, proportion, and representativeness of inpatient wards and outpatient clinics that appointed an ICLN	Total number of inpatient wards and outpatient clinics that appointed one or more link nurses ICLN meeting attendance; proportion and average for inpatient wards and for outpatient clinics Facilitators and barriers to participate and attend meetings	Project documents Project documents Interviews
**Effectiveness**	The (perceived) impact of the program	Change over time in compliance with the hospital dress code Change over time in compliance with hand hygiene protocol Empowerment of link nurse Perceived impact of link nurse activities on infection prevention policies (e.g. hand hygiene and dress code) Perceived mechanisms that contributed to this impact Perception of ward management regarding the skills of link nurses to disseminate their knowledge and influence routine practice	Details published elsewhere [20] Direct observations Psychological Empowerment Instrument Interviews Interviews Ward management survey
**Adoption**	The willingness of stakeholders to participate in the implementation of the link nurse program The willingness of ICLN to initiate link nurse activities	Stakeholders that were involved in the start of the link nurse project Work engagement of link nurses Willingness and motivation of link nurses to initiate link nurse activities Factors that influence willingness and motivation of link nurses	Project documents UWES 9 –questionnaire Interviews Interviews
**Implementation**	The fidelity of the ICLN program (was it implemented as intended?)	Implementation process, preconditions and contextual factors Use of the program by link nurses	Project documents Interviews
**Maintenance**	Did the ICLN program become part of the hospital or department routine?	Routines and policies at hospital level that include link nurses Link nurse activities that became part of the routine at the ward level	Project documents Interviews

#### Project documentation

During the project we registered the development of the program, information on program outline, stakeholders that were involved, education and training sessions, meeting attendance, and ward-based plans of action.

#### Direct observations

Since 2014, compliance with the hand hygiene protocol was measured for all inpatient wards once a year. Infection control practitioners and students were trained according to the WHO’s Hand Hygiene Technical Reference Manual and Training Films (https://www.who.int/teams/integrated-health-services/infection-prevention-control/hand-hygiene/training-tools). Observations were performed unannounced and discrete, but not covert [21].

Between March 2014 and June 2016, health care workers were observed for adherence to the hospital dress code in hospital hallways. Observers noted the type of HCW and scored each item of the dress code. ‘Compliant with the protocol’ was defined as adherence to all items [20].

#### Questionnaires

Empowerment of ICLN was measured in 2017 and 2018 by the Psychological Empowerment Scale (PES) [22]. This 12-item, 7-point Likert scale survey measures a motivational construct based on four subscales: meaning (the value of the ICLN role in relation to the link nurses’ ideals and beliefs), competence (self-efficacy / belief in his or her capability to perform link nurse activities), self-determination (the link nurses’ sense of having a choice in initiating and regulating actions) and impact (the degree to which a link nurse can influence the implementation of infection prevention policies in their own ward). Scores reflect how much individual link nurses wish to shape their role and to implement infection prevention policies in their ward, higher scores representing more determination. The validity of the PES has been established before; Cronbach alpha reliability ranged between 0.85 and 0.91 for total psychological empowerment [23].

Engagement of ICLN was measured in 2017 and 2018 using the nine-item Utrecht Work Engagement Scale (UWES-9) [24]. This 9-item, 7-point Likert scale survey measures 3 components of work engagement: vigor (feeling strong and resilient in the role of ICLN), dedication (commitment to being a link nurse), and absorption (merging with the role of the link nurse in a positive way). Higher scores represent a higher level of work engagement. The UWES has been validated in several countries, and has reasonable construct validity and high reliability (*⍺* = 0.93) [24–27].

Link nurses were asked to answer both questionnaires with the ICLN role in mind and to fill in their years of work experience.

In 2017, a survey among ward managers was performed to collect data on their perception of the skills and impact of ICLN. Three questions with a 5 point Likert scale measured if ward managers perceived their link nurses as skilled, proactive in performing their role and impactful. An open text box provided the opportunity for additional comments.

#### Interviews

Semi-structured face-to-face interviews were performed between April 2 and June 25, 2019 to capture and understand personal views and experiences of ICLN. These interviews, with link nurses that participated in our ICLN program were part of another qualitative study. The study aims aligned with the aim of the current study, which made secondary data analysis possible. For this study all participants provided a written consent.

### Data analysis

To evaluate this ICLN program structured the descriptive, quantitative and qualitative data and organized the outcomes along the five RE-AIM dimensions: Reach, Effectiveness, Adoption, Implementation and Maintenance.

#### Quantitative analysis

The number and proportion of wards that appointed one or more ICLN and the number, proportion and average of meeting attendance per ward (Reach) were calculated.

The results of the direct observations were used to calculate change over time for hand hygiene (Effectiveness). The adherence to the hospital dress code was published elsewhere [20].

The ward management survey and background questions were analyzed using descriptive statistics (Effectiveness). Cronbach's alpha was calculated to establish the reliability of the PES and UWES in the study sample. Measures of central tendency (median) and variability (inter quartile range) were used to describe scores for engagement to the link nurse role (Adoption). Spearman's correlations of work experience with engagement and empowerment were calculated. All data was analyzed using R Studio version 4.0.3 (R Foundation for Statistical Computing, Vienna, Austria).

#### Qualitative analysis

For this study, interviews were reanalyzed with direct content analyses. Pre-determined codes were based on results of other studies on ICLN and on the five dimensions of the RE-AIM framework. Interpretation of text segments and codes were discussed by the research team. The codes that were used can be found in Additional file 2. All data was analyzed in Atlas.Ti software version 8.0 for Windows.

Project documents were systematically searched for information on the implementation process, preconditions and contextual factors, stakeholders that were involved and new routines and policies that include ICLN (Implementation, Maintenance).

#### Data synthesis

The project documents provided data for all five of the RE-AIM dimensions. First, we summarized findings from these documents per dimension. Next, the outcomes of the interview data were added. Evidence from the remaining data sources was then integrated where applicable.

To explore how the program impacted link nurses and IPC routines, we looked at the domains Effectiveness, Adoption and Implementation, and to explore how the program impacted routines specific to our university hospital we looked at the dimensions Reach and Maintenance.

## Results

### Reach

At the start of the program, all 25 inpatient wards of our hospital appointed at least one ICLN. Some of these wards choose to appoint two link nurses, to assure continuity. In the following years the number of wards that participated declined to 20 wards. None of the inpatient wards dropped out of the program for longer than one year. The number of outpatient clinics that appointed an ICLN fluctuated per year between eight and fourteen. Some wards struggled with high turnover of staff and with timely replacement of their link nurses. Overall, the average meeting attendance per ICLN per year was 61% for inpatient wards and 34% for outpatient clinics (Table 2).

**Table 2:** Reach of the ICLN program

	**2014**	**2015**	**2016**	**2017**	**2018**	**overall**
	**n(%)**	**n(%)**	**n(%)**	**n(%)**	**n(%)**	**%**
Inpatient wards that appointed an ICLN	25(100)	24(96)	23(92)	22(88)	20(80)	91.2
Outpatient clinics that appointed an ICLN	10(56)	14(77.8)	9(50)	11(61.1)	8(44.4)	57.8
	**mean(%)**^**‡**^	**mean(%)**^**‡**^	**mean(%)**^**‡**^	**mean(%)**^**§**^	**mean(%)**^**‡**^	**%**
Meeting attendance per inpatient ward	2.7(67.5)	2.7(67.5)	1.9(47.5)	3.8(63.3)	2.4(60)	61.2
Meeting attendance per outpatient clinic	0.9(22.5)	1.2(30)	0.7(17.5)	1.5(37.5)	2.4(60)	33.5

Four meetings per year were held (^‡^*n* = 4). In 2016, six meetings were held (^§^*n* = 6)

In interviews, ICLN reported feeling facilitated to participate in the program due to their role as a senior staff nurse. Less active ICLN felt their ward managers failed to prioritize infection prevention. Time was also mentioned as a barrier to attend meetings, especially by ICLN from outpatient clinics.

*My manager does not encourage me to attend meetings, I don't think infection prevention is on the management agenda right now. [interview 1, outpatient clinics]*

### Effectiveness

The ICLN program focused on promoting compliance with infection prevention measures, such as the hand hygiene protocol or hospital dress code. Details on the role of the link nurse program in the uptake of the hospital dress code were published elsewhere [20]. During meetings the technique of hand hygiene and the identification of the World Health Organization’s five moments of hand hygiene were discussed. ICLN learned how to observe compliance and how to collaborate with the ward manager to improve practice. We observed an absolute increase in compliance of 26,5% in four years from 44.5% [95%CI, 42.9–46.0] in 2014 to 70.9% [95%CI, 69.4–72.4] in 2018.

The PES results revealed that link nurses felt moderately to highly empowered (e.g. able to meet the demands of the link nurse role) with a median total score of 5.0 (IQR 4.8–5.4) in 2017 and 5.2 (IQR 4.8–5.4) in 2018 (Table 3). Empowerment of the link nurses did not correlate with work experience (*r* 0.09, *p* = 0.51).

**Table 3:** Scores on the psychological empowerment scale (PES)

	**2017**		**2018**	
	**Median(IQR)**	**⍺**	**Median(IQR)**	**⍺**
Work experience in years	8 (5–22)		15 (5.5–33.8)	
Total PES score	5.0 (4.8–5.4)	.83	5.2 (4.8–5.4)	.94
Meaning	5.7 (5.3–6.0)	.58	5.7 (5.0–6.0)	.92
Competence	5.0 (4.7–5.7)	.70	5.3 (5.0–5.7)	.85
Self-determination	4.7 (4.3–5.0)	.68	5.0 (4.7–5.7)	.86
Impact	4.7 (4.0–5.0)	.70	4.3 (4.2- 5.0)	.92

Response rates were 86.0% (*n* = 37) for 2017 and 44.2% (*n* = 19) for 2018. *⍺* = Cronbach’s alpha *IQR* interquartile ranch

From the interviews it transpired that ICLN perceived their contribution to the improvement in guideline adherence as pivotal. They felt that the knowledge and skills that they had learned and tools that were provided by the program contributed to their link nurse activities. Working side by side with their peers enabled ICLN to observe non-compliance, to provide direct feedback and to provide them with solutions to overcome barriers (e.g. suggest placing extra dispensers).

*You have to keep repeating the observations. At first we missed disinfection of our hands at one specific moment over and over again. We presented the results of our observations to our colleagues and during the staff meeting. We are currently planning our next observations. [interview 8, outpatient clinics]*

*I followed a workshop on how to provide constructive feedback … After this workshop I realized I was communicating a bit too direct... So, because I was trained in measuring hand hygiene and in providing feedback to my peers I gained expertise in observing and dealing with non-compliance. [interview 9, outpatient clinics]*

In contrast, being the only nurse in a multiprofessional team working in an outpatient setting made it more complicated to take action.

*With many different disciplines working in our outpatient clinic there might be different needs, too. I can't oversee all of these needs. [interview 1, outpatient clinics]*

Surveyed ward managers underpinned the impact of their link nurses on infection prevention practices and valued them with a median score of 4 (IQR 3–4) out of 5 (Table 4). Ward managers felt that continuing to monitor the impact of their actions was harder for link nurses than to initiate improvements and that the support of the ward management was pivotal in dealing with resistance and in sustaining the effects.

**Table 4:** Perceived impact of link nurses by ward management

	**Median (IQR)**
The link nurse at my department has sufficient knowledge about infection prevention measures and sufficient skills to implement them	4 (4–4)
The link nurse at my department disseminates knowledge on infection prevention	4(3–4)
The link nurse at my department has sufficient impact on compliance with infection prevention measures	4(3–4)

### Adoption

The board of nursing directors adopted the ICLN program and committed to the recruitment of ICLN through the ward managers. Program principles were highly valued at the organizational level and the program was used as a blueprint for link nurse programs in other disciplines. Partnerships were established with leaders of other programs, such as medication safety, programs for the frail elderly and programs for wound care. The project was awarded the hospital’s Profile Prize 2016, which is awarded annually by the board of directors to multi-disciplinary teams that have delivered exceptional multi-year performances in one or more core tasks of the hospital.

In 2017, the link nurses perceived themselves as highly engaged with the link nurse role, with a median work engagement score of 5 (IQR 4.1–5.3). This dedication sustained during 2018; ICLN reported a median score of 5.1 (IQR 4.9–5.6) (Table 5). Work engagement of the link nurses did not correlate with work experience (*r* 0.17, *p* = 0.20).

**Table 5:** Scores on the Utrecht Work Engagement Scale (UWES)

	**2017**		**2018**	
	**Median(IQR)**	**⍺**	**Median(IQR)**	**⍺**
Work experience in years	8 (5–22)			
Total UWES score	5 (4.1–5.3)	.90	5.1 (4.9–5.6)	.96
Vigor	5 (4.0–5.3)	.75	5 (5.0–5.3)	.91
Dedication	5 (4.3–5.7)	.85	5.4 (5.1–6.0)	.92
Absorption	5(3.7–5.3)	.76	5 (4.4–5.6)	.94

Response rates were 86.0% (*n* = 37) for 2017 and 51.2% (*n* = 22) for 2018

*SD* standard deviation, *IQR* interquartile ranch ⍺ = Cronbach’s alpha

Factors that promoted work engagement were the commitment of the hospital and the ward manager to the program and the availability of the infection control practitioner.

*I feel supported by the organization ... I am so proud of our achievements. When I want to implement something, I get carte blanche from my ward manager. The team is also open to improvement. We have developed a positive team spirit … The infection control practitioners are very accessible, visible within the organization and they keep their promises. That is what keeps my commitment to the ICLN role. [interview 4, clinical ward]*

### Implementation

At first the program focused on education of ICLN and goals at the hospital level. Meetings were held four times a year. ICLN where educated on predetermined infection prevention topic, trained to perform quick scans and to report their findings to their peers.

The program was adapted to fit the needs of ICLN and was based on ongoing evaluation with ICLN, ward management and the board of nursing directors. Meetings evolved from exchange of knowledge to training of skills during simulation and implementation sessions. We collaborated with specialists in education and training, implementation, project management, personal effectiveness and leadership skills to prepare trainings sessions. During these sessions, time was also dedicated for ICLN to share their best practices.

In addition to hospital-wide infection prevention priorities (e.g. hospital dress code, hand hygiene), goals were formulated per ward (e.g. the proper use of non-sterile gloves, hygiene during vena puncture) together with the ward management, ICLN and the infection control practitioner. These plans also described the tasks and responsibilities for the ICLN, the ward manager and the infection control practitioner and the scheduled moments to discuss the progress of the plan. This way a bespoke plan of action was created to fit the local context and infection prevention issues of each ward. ICLN felt these plans helped to structure their tasks.

*In my department we work as a team, the ward manager, a physician, an infection control practitioner and two ICLN. We have made an annual plan and discuss the progress of this plan, the barriers that we encounter and divide upcoming tasks during monthly meetings. This way we discuss the infection prevention topics that we feel needs to be addressed in our ward and it provides structure for our actions. [interview 8, inpatient ward]*

### Maintenance

The ICLN program was described as a key component of the infection prevention policy and the link nurse role was incorporated in the Infection Prevention Plan of the hospital. This plan described the organization and aims of infection control and prevention at the hospital level. Since its effectuation it facilitated and guided activities of the Hospital Infection Prevention Committee in supervising and coordinating infection prevention policies and reporting to the executive board.

ICLN on all inpatient wards were trained by infection control practitioners; the training continuously offered to refresh skills or to train new ICLN. ICLN continued to measure hand hygiene compliance on a regular basis. Additionally, ICLN were taken up in the list of stakeholders in outbreak management, and were assigned a key role in the dissemination of local outbreak information at the ward level.

The role of the link nurse and outcomes of the ward-based plans of action were discussed by ward managers and nursing directors during quarterly meetings. Ward managers were committed to provide the ICLN sufficient time to observe hand hygiene practices.

## Discussion

In this study we evaluated an infection control link nurse program that was developed in our academic hospital; this evaluation was performed according to the RE-AIM framework. The results contribute to the understanding of the relation between how an ICLN program is implemented and its sustained effectiveness [3, 28]. Strong reach on the clinical wards and adoption of the program at hospital and individual level was observed; this effect appeared to be the result of endorsement of the program by hospital and ward management and of collaboration with other stakeholders. Implementation was facilitated by providing enough time for adoption of the program. Flexible implementation with adjustments of the program, based on ongoing evaluation led to a shift of focus from hospital goals to goals tailored to the ward level. Our program had low reach and therefore low impact in the outpatient clinics.

Strong adoption of the program by hospital and ward management highlights the well-known importance of support for effective implementation of safe practices. A ward manager that supports and inspires staff to excel and forms partnerships across disciplines is more likely to succeed in the implementation of safe practices [29, 30]. Saint et al*.* stated that infection control practitioners also have an important leadership role in implementing safe practices; positive interactions with staff, enthusiasm and communicating the end goals of the program are powerful skills [31]. Thus, these behaviors should be kept in mind when leading a link nurse program.

Psychological empowerment of the link nurses and their engagement likely contributed to the adoption and success of our program. Psychological empowerment of nurses is associated with the provision of safe care [32]. Empowerment of nurses correlates positively with work engagement [33, 34]. When nurses feel dedicated to their work, they are more willing to do well. Work engagement is associated with improved patient-quality outcomes and strong interdisciplinary collaborations [32–35]. In line with these findings and related to infection prevention, Gilmartin and colleagues found that greater job satisfaction of nurses correlated with a decreased risk of central line–associated bloodstream infections [35]. Ambiguous results were found regarding the assumption that link nurses should be experienced nurses to have authority, a precondition to have impact. Although being a senior staff nurse was mentioned by ICLN as a facilitator for the uptake of the role, our study could not demonstrate a correlation between work experience and empowerment nor between work experience and engagement. To actively engage and empower ILCN, we introduced a PDCA cycle at the ward level. This provided the opportunity to develop and implement infection prevention activities that were tailored to the local context and to strengthen inter-professional collaboration [30, 36]. It is known that local goals can differ from formal program goals; adaptation to the local context can facilitate success [37]. By co-developing interventions and monitoring implementation, we added to the infrastructure that was required for the implementation of the link nurse role at the ward level. By adding this component, our program progressively evolved to a multi-modal strategy as propagated by the World Health Organization. Interventions that include several elements have proven to be more effective when applied in an integrated way [38].

Our program had relatively low reach in the outpatient clinics. Link nurses from outpatient clinics in our study mentioned, apart from the lack of management support, a lack of resources as a barrier to take action, as observed in other studies [39]. In order to be effective, an infrastructure that allows ICLN to monitor practices and to be a role model in adhering to IPC measures is required. As in outpatient clinics the work of health care workers is more individually organized, it might be harder to be actively involved in supporting colleagues [40]. Due to the multiprofessional nature of teams working in the outpatient setting, and due to the setting, relationships between the various professionals are more hierarchical; this may hamper the role of the ICLN too. *Romijn *et al. found discrepancies in the perceptions of several groups of health care workers on interprofessional collaboration, especially between physicians and other professionals (e.g. nurses). These discrepancies were most apparent with respect to the discussion of new practices and sharing of opinions [41]. In this case situational leadership is needed rather than hierarchical leadership, which underpins the importance of training ICLN in soft skills and especially in leadership skills, as an essential element of ICLN programs [7].

Although case study results have limited generalizability, they yield the unique opportunity to evaluate interventions in their natural context. Key strengths of this evaluation included the use of multiple data sources and of a robust evaluation framework. It provides rich information on strategies to implement and sustain link nurse programs in acute care hospitals.

The main limitation of this study is the absence of formal and systematic data collection. For example, baseline PES and UWES scores were not assessed. We therefore cannot link engagement and empowerment of the link nurses to our program activities. Potentially, link nurses entered the link nurse program because of their work engagement, their interest in infection prevention and their enthusiasm and competences to engage in program activities. The secondary analysis of the interviews was done by only one researcher. This limitation was partially addressed by team discussions with all authors that were also involved in the primary analyses; the data was discussed on multiple occasions. During these sessions reflexivity was encouraged. It reduced the risk of interpretation of the data being guided by team members’ knowledge, experiences and expectations [42]. Selected quotations from participants are included to allow the reader to judge interpretations and credibility of the analysis.

While we show here that an ICLN program can reach and influence ICLN, and can help to improve safe practices, especially in inpatient wards of acute care hospitals, it is possible that we did not capture all specific pitfalls and benefits of our program. A prospectively designed evaluation of this program in multiple organizations may be needed to showcase the true values of ICLN programs. Patient care shifts from inpatient care to the outpatient environment, leading to more complex care and treatment of more vulnerable patients in outpatient clinics. Increased attention for infection prevention in this part of hospitals is therefore important [40, 43]. Future research should also investigate the possibilities to extend program activities to the outpatient setting.

## Conclusions

The adaptability of link nurse programs allows the development of program activities that fit the needs of ICLN and allows to tailor program goals and to align these with the goals specific to each ward. Context specific plans of action can be an appropriate and valuable addition to ICLN programs. This implementation approach was found valuable to provide the program with reach, and to make it effective, adoptable, and maintainable.

## Supplementary Information

**Additional file 1.** StaRI-checklist.

**Additional file 2.** Interview codes.

## Data Availability

The datasets used and/or analyzed during the current study are available from the corresponding author upon reasonable request.

## Abbreviations

ICLN: Infection control link nurse
IPC: Infection prevention and control
UWES-9: Utrecht Work Engagement Scale
PES: Psychological Empowerment Scale
StaRI: Standards for Reporting Implementation Studies
SQUIRE 2.0: Revised Standards for Quality Improvement Reporting Excellence
