# Supplementary material for: Evaluation of an infection control link nurse program: an analysis using the RE-AIM framework
Source: BMC Health Serv Res. 2023 Feb 9;23:140. doi: 10.1186/s12913-023-09111-5 (PMC9912654; doi:10.1186/s12913-023-09111-5)
Supplement: Supplementary file 2 — Additional file 2. Interview codes. [file 12913_2023_9111_MOESM2_ESM.docx]

**ADDITIONAL FILE 2 -**  **Pre-determined interview codes**

| Facilitators and barriers to participate and attend meetings |
| --- |
| Perceived impact of link nurse activities on infection prevention policies (e.g. hand hygiene and dress code) |
| Willingness and motivation of infection control link nurses to initiate link nurse activities |
| Factors that influence willingness and motivation of infection control link nurses |
| How infection control link nurses used the program |
